# Tomato flu outbreak in India: Why is it an impending public health emergency?

**DOI:** 10.1097/JS9.0000000000000032

**Published:** 2023-05-09

**Authors:** Anushree Rai, Olivier Uwishema, Aderinto Nicholas, Mortada Abbass, Lama Uweis, Sara Arab, Rayyan El Saleh, Irem Adanur, Daniel Stephen Masunga, Abubakar Nazir

**Affiliations:** a Oli Health Magazine Organization, Research and Education, Kigali, Rwanda; b Chhattisgarh Institute of Medical Sciences, Bilaspur, Chhattisgarh, India; c Clinton Global Initiative University, New York, USA; d Faculty of Medicine, Karadeniz Technical University, Trabzon, Turkey; e King Edward Medical University, Lahore, Pakistan; f Department of Medicine and Surgery, Ladoke Akintola University of Technology, Ogbomoso, Nigeria; g Faculty of Medicine, Beirut Arab University, Beirut, Lebanon; h Kilimanjaro Christian Medical University College (KCMUCo), Moshi, Tanzania

*Dear Editor*,

Highlights

- Tomato flu virus was first identified on 6th May 2022 in the Kollam District of Kerala and later spread to the Indian states of Haryana, Odisha, and Tamil Nadu. Tomato flu has been proposed to be a clinical variant of hand, foot, and mouth disease (HFMD) and is caused by Coxsackievirus A-6 and A-16 of the group Enterovirus.
- It is essential to properly isolate known or suspected cases for 5–7 days after symptom onset and take additional preventative measures to stop the tomato flu virus from spreading to other regions of India.
- Investigations should be made promptly to take action in the event of an outbreak^11^. Any samples of the respiratory, fecal, or cerebrospinal fluid (in cases of encephalitis or brain inflammation) must be taken within 48 h of the disease.

The novel tomato flu infection was first identified in Kerala, India, on 6th May 2022^1^. It is proposed to be related to hand, foot, and mouth disease (HFMD) and is caused by Coxsackievirus A-6 and A-16 of the Enterovirus group. The disease is so named due to the eruption of red-colored painful blisters that resemble tomatoes^1,2^. These blisters gradually increase in size and resemble the blisters in monkeypox patients^1^. According to *The Lancet Respiratory Medicine Journal*, by 26th July 2022, 82 children from the Kollam district of Kerala were infected with this virus^1^. Additionally, some cases have been reported from other states, namely Odisha, Haryana, and Tamil Nadu^1^. It is stated that children, especially young ones (1–5 years old) and immunocompromised adults are the target population for this infection^1^. The route of transmission of the virus is thought to be through close contact, especially by touching infected surfaces^1^. Tomato flu causes symptoms that resemble those of several other viral infections, like the novel coronavirus disease 2019 (COVID-19) and chikungunya^1^. These symptoms include painful blisters, fever, myalgia, swelling of joints, and dehydration^1^. Thus, before confirming the diagnosis, several serological and molecular tests are done to rule out other infections^1^. Once the diagnosis is established, patients are isolated for 5–7 days and recommended to have plenty of fluids in addition to bed rest and symptomatic treatment^1^. The infection has no definitive treatment, but the vast majority of cases were self-limiting without any life-threatening sequelae^1,3^. Rarely does it leave some complications like muscle paralysis and encephalitis^3^.

## Epidemiology of tomato flu in India

**Figure 1:**
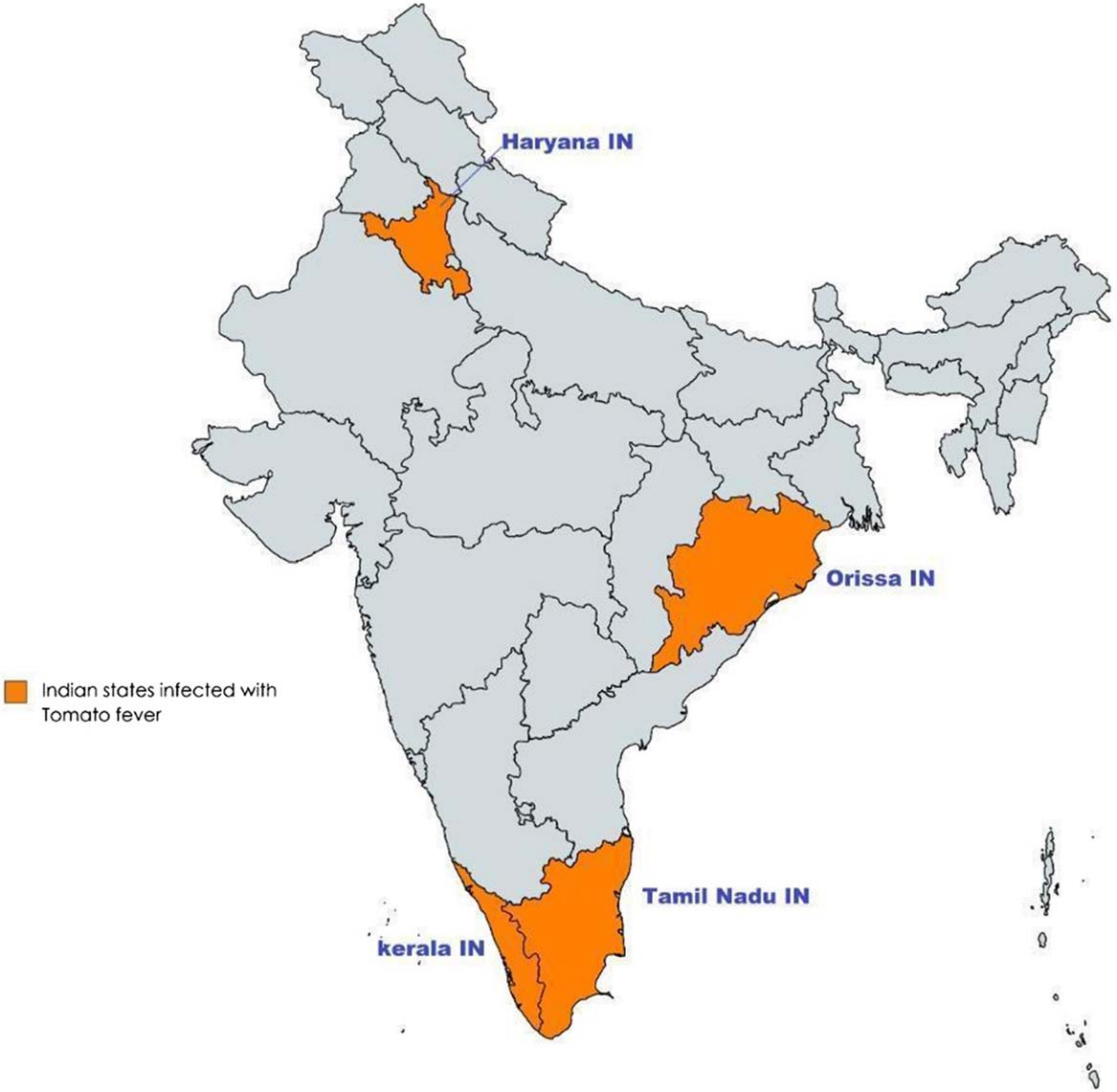
A map showing the Indian states where most cases of tomato flu were identified.

An outbreak of a rare viral disease referred to as ‘Tomato Flu’ has hit India’s southern cities (see Fig. 1), leaving more than 100 cases of infected children, mostly below the age of 10 years^4,7^. On 6 May 2022, the Healthcare Department of the Tamil Nadu Government declared that the contagious tomato flu had spread to the states of Kerala, Odisha, and Haryana. Kerala was first hit by this virus in 2007, and was speculated to be an aftereffect of chikungunya disease and food poisoning cases which were retrospectively diagnosed as tomato flu^4,6,8^. Tomato flu is predominantly detected in children as they are at higher risk of coming in direct contact with infected children while sharing utensils, toys, and clothing and touching unclean surfaces^7^. This new virus has led to the eruption of rashes and blisters similar to that found in HFMD^2,5^. The disease was so named after the tomato-like large red blisters that appear on the skin of affected children, similar to monkeypox blisters^7,9^ (see Table 1). Symptoms usually start as a mild fever and poor appetite with a sore throat^4,7^. Furthermore, the most noticeable symptoms of the disease include a rash with fever, joint pain, and swelling, as well as typical flu-like symptoms like fatigue, nausea, vomiting, diarrhea, dehydration, and body aches^6,7^. Samples for testing are usually taken from stool or from the throat to be tested by rapid antigen testing^7^. Doctors have recommended isolation of affected children for 5–7 days after the onset of symptoms and immediate hospitalization in severe cases^7^. According to a senior health official in the Delhi government, the prevalence of chikungunya and dengue viruses has led to weaker immune systems in children and left them highly vulnerable to the tomato flu disease^4,10^.

**Table 1:** Difference between tomato flu and monkeypox disease.

	Tomato flu	Monkeypox
Causative agent	Coxsackievirus A-6, A-16	Monkeypox virus (Orthomyxovirus)
Prevalence in kids	High	Low
Rashes (characteristics)	Red painful fluid-filled blisters (that look like tomato)	Red painful flat bumps Phases: macules, papules, vesicles
Mode of transmission	Direct contact Lack of hygiene	Animal to human–zoonotic, Human–human: respiratory secretions and droplets
Symptoms	Fever, joint pain and swelling, body ache, and fatigue, followed by rashes	Fever, chills, swollen lymph nodes, muscle aches, headache, respiratory symptoms
Incubation period	5–7 days	3–17 days
Treatment	Not yet available	Not yet available

## Etiology of tomato flu

The published report of test findings of children infected with tomato flu had a travel history to the United Kingdom, where they had played with a kid who had recently contracted ‘tomato flu’^3^. A week after returning, the 13-month-old girl and her 5-year-old brother developed a rash consisting of small fluid-filled blisters with no other symptoms^3^. According to research by Julian Tang of the University of Leicester in the United Kingdom and his colleagues, the children had a coxsackievirus infection, which is what causes HFMD^3^. It is likely that HFMD, a common and typically mild illness in children, is causing the so-called tomato flu in India^3^. On that note, the name ‘tomato flu’ is a misnomer because coxsackieviruses, a member of the enterovirus family, are unrelated to influenza viruses and have no connection to plants^3^. It is also conceivable that some of the illnesses are caused by the dengue and chikungunya viruses spread by mosquitoes^3^. However, the symptom that gave rise to the phrase ‘tomato flu’, the characteristic painful red fluid-filled blisters, is not a symptom of either dengue or chikungunya^3^. In another frame of reference, some physicians in Kerala believe it to be a new illness^3^. Nevertheless, according to Tang, there can be significant diversity in the rashes brought on by a single infection^3^. In addition, new enterovirus strains have recently spread over the world from China, so the manifestations of these more recent lineages can vary^3^. Coxsackievirus A-6 and A-16 are the main causes of the current instances of HFMD in India, and there has reportedly been an increase in HFMD infections since kids were allowed to go back to school after the COVID-19 ban was lifted^11^. Another possible cause of the sickness is Enterovirus 71, but it currently has a low prevalence^11^. Moreover, the increased vigilance for this viral infection could be due to an actual increase in the number of cases or as a part of a lesson learned from the COVID-19 outbreak response^11^.

## Current efforts to mitigate tomato flu in India

In recent days, the tomato flu outbreak in India has gathered global attention. Cases have been detected in four countries and the accelerating rate of the spread of this outbreak has been a source of concern for both local and international health agencies^12^. Although tomato flu infections have existed in India for nearly 2 decades, the recent number of cases has been more than the country’s normal count^12^. The exact cause of the increase in cases has not been pinpointed but many have attributed it to the aftermath of the COVID-19 pandemic and the reopening of schools in India^12^. While the World Health Organization (WHO) has not issued an international stance, Indian health authorities have put necessary measures in place to curb the outbreak^12^. The Indian health authority has increased health surveillance and has recommended swift testing to ensure an adequate response to the outbreak. Lessons from the COVID-19 pandemic have shown that swift actions are instrumental in mitigating the impact of endemic disease outbreaks^12,13^. Since there are no vaccines or drugs for the management of the tomato flu, most of the infected cases are treated symptomatically^12^. The most effective way to prevent further spread of the infection is to isolate the suspected cases for 5–7 days from the onset of symptoms^12^.

## Recommendations

**Figure 2:**
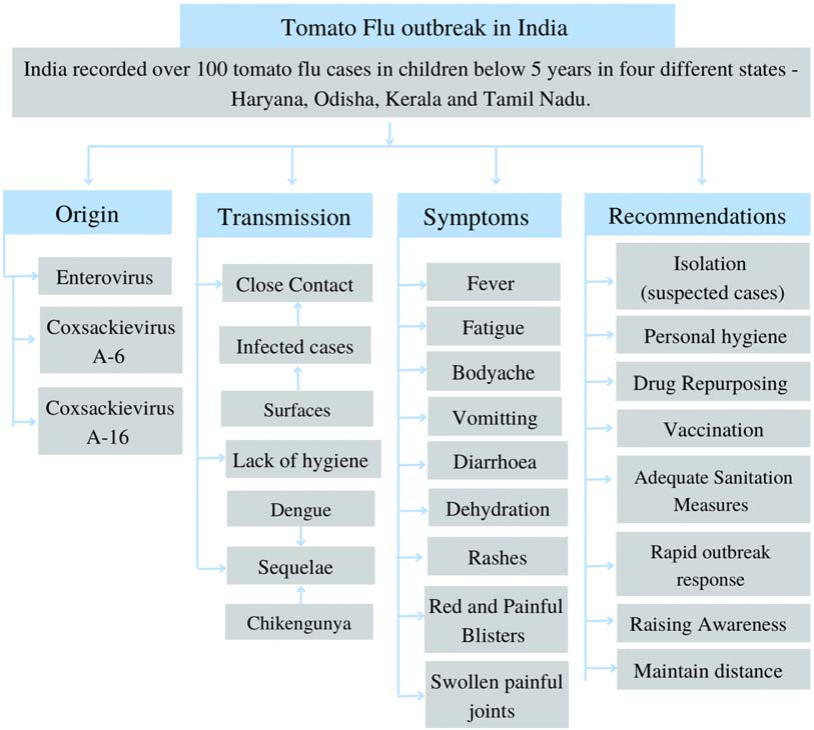
Summarize tomato flu in India: origin, transmission, symptoms and recommendations.

Like other influenza strains, tomato flu is extremely contagious^1^. Therefore, it is essential to properly isolate known or suspected cases for 5–7 days after symptom onset and take additional preventative measures to stop the tomato flu virus from spreading to other regions of India^1^ (see Fig. 2). The most effective and economical methods for protecting the public from viral infections, particularly in children, the elderly, immunocompromised individuals, and those with underlying medical conditions, are drug repurposing and immunization^1^. Tomato flu cannot yet be treated or prevented with antiviral medications or vaccinations^1^. And to understand the need for prospective treatments, additional follow-up and monitoring for significant outcomes and sequelae are required^1^. The investigations should be timely made to take action in the event of an outbreak^11^. Any samples of the respiratory, fecal, or cerebrospinal fluid (in cases of encephalitis or brain inflammation) must be taken within 48 h of the disease^11^. Such time restrictions do not apply to skin-scraping samples or lesions that are being biopsied^11^. The greatest method of prevention is maintaining good hygiene, sanitizing the immediate area, and keeping the infected child from sharing toys, clothes, food, or similar objects with other children who are not ill^1,14^. The Center’s Advisory to the state, which was released on Tuesday, emphasized adequate preventive measures in infants^11^. According to the advice, anyone who exhibits symptoms of the infection should stay isolated for 5–7 days^11^. Kids should be warned about the infection and told to avoid hugging or touching kids who have fevers or rashes^11^. If a child exhibits symptoms, they should be kept apart, their equipment, clothes, and bed must be routinely sanitized, they should be properly hydrated, and the blisters must be washed with warm water^11,15^. Parents should urge their kids to practice good hygiene, avoid thumb or finger sucking, and use a napkin if they have a runny nose^11^.

## Conclusion

The highly contagious viral infection, tomato flu, was first identified on 6th May 2022, in the Kollam District of Kerala. And as of 26th August 2022, more than 100 tomato flu cases of children below 5 years of age have emerged in the Indian states of Haryana, Odisha, Tamil Nadu, and Kerala.

It has been proposed to be a clinical variant of HFMD – a common infectious disease that targets children between 1 and 10 years and immunocompromised adults. It is caused by the Coxsackievirus A-6 and A-16 of the group enterovirus. The most noticeable symptoms of the disease include a rash with fever, joint pain and swelling, as well as typical flu-like symptoms like fatigue, nausea, vomiting, diarrhea, dehydration, and body aches. It has also been highlighted that the spread of chikungunya and dengue viruses left Indian children vulnerable to the tomato flu disease due to a weaker immune system.

The Indian health ministry has issued increased health surveillance to curb this outbreak. With no vaccines and symptomatic treatment, isolation and swift testing have become the most important measures to prevent further spread. To help contain the ongoing crisis, certain recommendations for individuals and governments like routine sanitization, parental advisory, additional follow-up and monitoring of significant outcomes have been made. Emphasis has been further laid on research in prospective treatments and vaccination to understand and control the extremely contagious tomato flu.

## Ethical approval

Not applicable.

## Sources of funding

Not applicable.

## Author contribution

O.U.: conceptualization, project administration, and writing – review and designing. All authors contributed to manuscript writing and data collection and assembly and were involved in the final approval of the manuscript.

## Conflicts of interest disclosure

There are no conflicts of interest.

## Guarantor

Abubakar Nazir, E-mail: abu07909@gmail.com. ORCID ID: 0000-0002-6650.

## References

[1] Chavda VP Patel K Apostolopoulos V . Tomato flu outbreak in India. Lancet Respir Med 2023;11:E1–E2. 35987204 10.1016/S2213-2600(22)00300-9 PMC9385198

[2] Zee News. Tomato fever grips Kerala, Tamilnadu, steps up vigil on border. Zee News. Accessed 24 August 2022. https://zeenews.india.com/india/tomato-fever-grips-kerala-tamil-nadu-steps-up-vigil-on-border-2463348.html#:%7E:text=The%20main%20symptom%20of%20tomato,diarrhea%20and%20nausea,%20and%20vomiting

[3] Page ML . A common childhood illness could be behind ‘tomato flu’ outbreak. New Scientist. Accessed 25 August 2022. https://www.newscientist.com/article/2335287-a-common-childhood-illness-could-be-behind-tomato-flu-outbreak/

[4] Mukherjee D Ruchika F Pokhrel NB . Tomato fever and COVID 19, a double hit in the Indian health system. Immun Inflamm Dis 2022;10:e677. 35894713 10.1002/iid3.677 PMC9274800

[5] Sayantani B . India logs 82 cases of ‘Tomato Fever’ in children below 5 years, Lancet issues alert. Accessed 24 August 2022. https://www.livemint.com/science/health/india-logs-82-cases-of-tomato-fever-in-children-below-5-years-lancet-issues-alert-11660992908375.html

[6] Sreeraman VR . ‘Tomato Fever’ Replaces Chikungunya in Kerala. Accessed 24 August 2022. https://www.medindia.net/news/tomato-fever-replaces-chikungunya-in-kerala-23631-1.htm

[7] Sharma P . Over 100 cases of Tomato Flu detected in children under 9 years of age, health ministry issues advisory. Accessed 24 August 2022. https://www.livemint.com/science/health/over-100-cases-of-tomato-flu-detected-in-children-under-9-years-of-age-health-ministry-issues-advisory-11661265237625.html

[8] Disease Alerts/Outbreaks reported and responded to by States/UTs through Integrated Disease Surveillance Program (IDSP). 2022). https://www.idsp.nic.in/index4.php?lang=1&level=0&linkid=406&lid=3689

[9] Firstpost. Tomato Flu Outbreak in India: understanding the contagious disease. 2022. https://www.firstpost.com/india/tomato-flu-outbreak-in-india-understanding-the-contagious-disease-11087761.html

[10] The Guardian. Tomato flu outbreak in India spreads to two more states. Amrit Dhillon. 2022. https://www.theguardian.com/world/2022/aug/23/tomato-flu-outbreak-in-india-spreads-to-two-more-states

[11] Dutt, A. & Explained Desk. Explained: What is tomato flu – and the enterovirus that may be causing the outbreak. The Indian Express. Accessed 25 August 2022. https://indianexpress.com/article/explained/everyday-explainers/explained-tomato-flu-enterovirus-outbreak-8107780/lite/

[12] Sudhakar K . Alert in Karnataka over tomato flu. The Hindu, 12 May 2022. Accessed 24 June 2022. https://www.thehindu.com/news/national/karnataka/alertin-karnataka-over-tomato-flu/article65405330.ece

[13] Uwishema O Frederiksen KS Correia I . The impact of COVID-19 on patients with neurological disorders and their access to healthcare in Africa: a review of the literature. Brain Behav 2022;12:e2742. 35951730 10.1002/brb3.2742 PMC9480907

[14] Sun J Uwishema O Kassem H . Ebola virus outbreak returns to the Democratic Republic of Congo: an urgent rising concern. Ann Med Surg (Lond) 2022;79:103958. 35757313 10.1016/j.amsu.2022.103958 PMC9218350

[15] Uwishema O Mahmoud A Wellington J . A review on acute, severe hepatitis of unknown origin in children: a call for concern. Ann Med Surg (Lond) 2022;81:104457. 36147181 10.1016/j.amsu.2022.104457 PMC9486726

